# Seasonal variations in renal biopsy numbers and primary glomerular disease features based on the Japan renal biopsy registry

**DOI:** 10.1038/s41598-023-32182-7

**Published:** 2023-03-29

**Authors:** Go Kanzaki, Nobuo Tsuboi, Takashi Yokoo, Noriko Uesugi, Kengo Furuichi, Akira Shimizu, Hitoshi Sugiyama, Hiroshi Sato, Hitoshi Yokoyama, Committee for Renal Biopsy and Disease Registry of the Japanese Society of Nephrology, Hiroshi Sato, Akira Shimizu, Hitoshi Sugiyama, Hiroshi Kitamura, Ritsuko Katafuchi, Shinichi Nishi, Motoshi Hattori, Ryohei Yamamoto, Toshiharu Ninomiya, Yoshihiko Ueda, Michio Nagata, Hirofumi Makino, Hitoshi Yokoyama, Shoji Kagami

**Affiliations:** 1 Division of Nephrology and Hypertension, Department of Internal Medicine, The Jikei University School of Medicine, Minato-ku, Tokyo, 105-8461 Japan; 2 Department of Pathology, Fukuoka University, Fukuoka, Japan; 3 Division of Nephrology, Kanazawa Medical University School of Medicine, Ishikawa, Japan; 4 Department of Analytic Human Pathology, Nippon Medical School, Tokyo, Japan; 5 Department of Human Resource Development of Dialysis Therapy for Kidney Disease, Okayama University Graduate School of Medicine, Dentistry and Pharmaceutical Sciences, Okayama, Japan; 6 Department of Internal Medicine, Sendai Hospital of East Japan Railway Company, Sendai, Japan; 7 Department of Clinical Pathology, National Hospital Organization Chiba-Higashi National Hospital, Chiba, Japan; 8 National Fukuoka Higashi Medical Center, Fukuoka, Japan; 9 Division of Nephrology and Kidney Center, Kobe University Graduate School of Medicine, Kobe, Japan; 10 Department of Pediatric Nephrology, Tokyo Women’s Medical University, Tokyo, Japan; 11 Health and Counseling Center, Osaka University, Osaka, Japan; 12 Department of Medicine and Clinical Science, Graduate School of Medical Sciences, Kyushu University, Fukuoka, Japan; 13 Department of Pathology, Dokkyo Medical University, Saitama Medical Center, Koshigaya, Japan; 14 Kidney and Vascular Pathology, University of Tsukuba, Ibaraki, Japan; 15 Department of Nephrology, Rheumatology, Endocrinology and Metabolism, Okayama University Graduate School of Medicine, Dentistry and Pharmaceutical Sciences, Okayama, Japan; 16 Department of Pediatrics, University of Tokushima Graduate School, Tokushima, Japan

**Keywords:** Nephrology, Kidney diseases

## Abstract

We analyzed the seasonal variations in the number of renal biopsies and clinical characteristics of primary glomerular disease in Japan using the Japan Renal Biopsy Registry (J-RBR). We retrospectively collected clinical and pathological data of patients with primary glomerular disease who were registered in the J-RBR between 2007 and 2018. Immunoglobulin A nephropathy (IgAN), minimal change nephrotic syndrome (MCNS), membranous nephropathy (MN), and postinfectious acute glomerulonephritis (PIAGN) constituted the four major glomerular disorders included in this study (total, 13,989; IgAN, 9121; MCNS, 2298; MN, 2447; and PIAGN, 123). The number of patients with IgAN or MCNS was higher during summer. However, no overt seasonal variations were observed in patients with MN or PIAGN. Subgroup analyses suggested that in the patients with IgAN, more renal biopsies of severe cases were performed during winter, probably owing to age and blood pressure. Furthermore, more renal biopsies of severe cases were performed during spring and winter in patients with MCNS even after adjusting for the abovementioned host factors. This study suggests that seasonal factors influence the decision to perform renal biopsy as well as the pathogenesis of primary glomerular disease. Thus, our findings may provide important insights regarding the pathophysiology of primary glomerular disease.

## Introduction

The association between seasonal factors and the onset and prevalence of various diseases have been previously investigated^1^. Reportedly, respiratory diseases^2^, hypertension^3^, and cardiocerebrovascular diseases^4,5^ tend to develop and worsen during winter, which is also when the rate of respiratory infection peaks^6^. Furthermore, cold weather alters physiological hemodynamics and hematological factors, thereby contributing to arterial thrombosis^7,8^. Consequently, acute kidney injury onset and dialysis introduction are also common during winter^9,10^.

The onset of glomerular disease is believed to be due to the interaction of genetic and environmental factors. Briefly, certain genetic factors predispose individuals toward an immune response that leads to glomerulonephritis, and inflammatory and noninflammatory immune mechanisms are considered to be involved in the pathogenesis of this glomerular injury^11^. Particularly, infectious and allergic diseases constitute the priamary external factors that influence the pathogenesis of lifetime diseases^12^. Group A beta-hemolytic streptococcus, a culprit pathogen of epidemic infections during winter, has been causally associated with acute glomerulonephritis^13^. The persistence of respiratory viruses may contribute to the development and progression of minimal change nephrotic syndrome (MCNS)^14^. Recent studies suggest that gut microbiota are involved in the pathogenesis of primary glomerular diseases, such as immunoglobulin A nephropathy (IgAN) and membranous nephropathy (MN). Gut microbiota are susceptible to environmental factors, such as personal habits and nutrition^15^. Additionally, children with idiopathic nephrotic syndrome exhibit a high incidence of allergic diseases, including atopic dermatitis and allergic rhinitis, and the number of cases is reportedly high during spring and autumn^16,17^. In adults, idiopathic nephrotic syndrome reportedly occurs more frequently during winter. Furthermore, IgAN, which is probably associated with viral infections and tonsillitis, tends to worsen during winter^18^. Patients with early diabetic nephropathy also exhibit considerably higher proteinuria and albuminuria during autumn and winter than during spring and summer, probably because of increased systolic blood pressure (BP) during winter^19^.

However, there have only been few studies regarding the relationship between primary glomerulonephritis and seasonal factors closely associated with the development of the infections and allergies, and to the best of our knowledge, there have been no such studies involving a large cohort of patients.

The Japan Renal Biopsy Registry (J-RBR) is a nationwide, multicenter, web-based, prospective renal biopsy registry established in 2007 to record clinical and pathological data of patients undergoing renal biopsy^20^. J-RBR data is particularly beneficial for analyzing the association of seasonal variations with disease pathogenesis, as Japan is a country with a homogeneous society that experiences four distinct seasons.

This study aimed to analyze the influence of seasonal variations on the number of renal biopsies and the clinical features of primary glomerular disease in Japan using the J-RBR and clarify the relationship between the various types of glomerulonephritis, which are closely related to external factors, and the seasons. We focused on the following four diseases: IgAN, MCNS, MN, and postinfectious acute glomerulonephritis (PIAGN), which are reportedly associated with allergies and infectious diseases and have a high incidence in Japan.

## Methods

### J-RBR system and patient selection

The J-RBR is a nationwide, multicenter registry system that was organized by the Committee for the Standardization of Renal Pathological Diagnosis and the Working Group for the Renal Biopsy Database of the Japanese Society of Nephrology in 2007^20^. Individual patient data, including basic patient information, clinical diagnosis, renal pathological findings, biochemical features, and urinalysis, were uploaded to the J-RBR website using the Internet Data and Information Center for Medical Research system of the University Hospital Medical Information Network (UMIN). The J-RBR is registered under the Clinical Trial Registry of UMIN (registration number, UMIN000000618). This study was approved by the Ethics Committee of the Japanese Society of Nephrology and conducted per the principles of the Declaration of Helsinki (Research of J-RBR in Japanese Society of Nephrology, No.79, J-RBR201904, September 2, 2019). Written informed consent was obtained from all the study participants or the parents if the participant was a child.

This retrospective study study included Japanese patients with primary IgAN, MCNS, MN, or PIAGN who were registered in the J-RBR from July 1, 2007 to January 1, 2018. The baseline characteristics of the patients, including clinical and pathological features at the time of the renal biopsies, were obtained from the J-RBR database.

**Figure 1:**
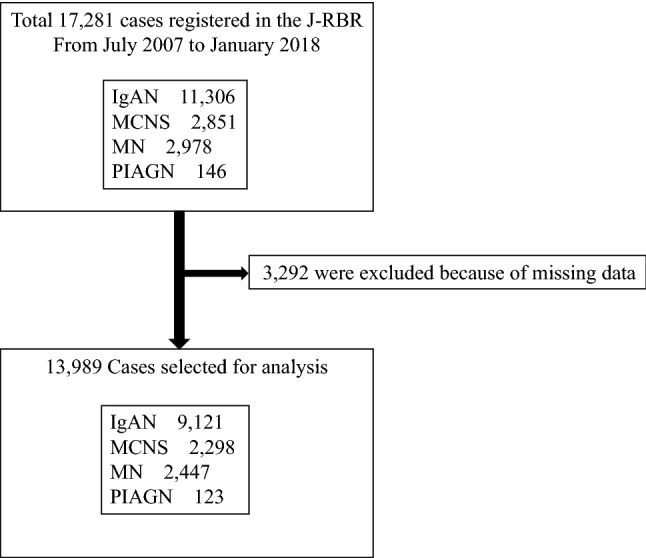
Patient selection. Extraction of patients with primary glomerulonephritis according to the Japan Renal Biopsy Registry (J-RBR), IgA nephropathy (IgAN), minimal change nephrotic syndrome (MCNS), membranous nephropathy (MN), and postinfectious acute glomerulonephritis (PIAGN).

During the registration period, 17,281 patients were registered in the J-RBR. Of these, 3292 patients were excluded because of missing data that were critical for the analysis. Overall, 13,989 patients (IgAN: 9121; MCNS: 2298; MN: 2447; and PIAGN: 123) were finally included in the analysis (Fig. 1).

Patients who were diagnosed with other renal or systemic diseases were excluded.

### Clinical measurements and definitions

The clinical data of the patienta, including age, sex, body mass index (BMI), systolic and diastolic BP, serum creatinine (sCr) levels, estimated glomerular filtration rate (eGFR), serum albumin levels, serum total cholesterol levels, and urinary protein excretion (UPE) rate, were evaluated. The eGFR was calculated using a three-variable equation modified for Japanese populations as follows: eGFR = 194 × age^−0.287^ × sCr^−1.094^ (× 0.739 if female)^22^.

The four seasons relevant to the climate in Japan were defined month-wise as follows: spring (March–May), summer (June–August), autumn (September–November), and winter (December–February)^23^.

To examine the possibility of applying the findings of this study in clinical practice, the ages of the patietns were classified into three categories: children (aged < 18 years), adults (aged 18–64 years), and older individuals (aged ≥ 65 years).

Hematuria was defined as more than five red cells per high power field (HPF) in urinary sediments and graded based on the number of red cells per HPF as follows: 0–4, 5–10, 11–30, and ≥ 30.

Based on the KDIGO 2012 guidelines modified for the Japanese population, the UPE rates at biopsy were classified as normal (< 0.15 g/day or g/gCr; A1), mild (0.15–0.49 g/day or g/gCr; A2), and severe (≥ 0.5 g/day or g/gCr; A3). Additionally, the eGFR at the time of biopsy was classified into five groups: G1, G2, G3a, G3b, G4, and G5 for ≥ 90, 60–89, 45–59, 30–44, 15–29, and < 15 mL/min/1.73 m^2^, respectively. According to these UPE rates and eGFR values, the chronic kidney disease (CKD) heat map classified renal prognosis as low, medium, high, and very high^24–26^.

### Statistical analysis

We first described the baseline characteristics of the entire study population and of the patients in the four groups (IgAN, MCNS, MN, and PIAGN). Continuous variables were presented as the mean and standard deviation or frequencies with percentages (in parentheses). Seasonal differences in the number of kidney biopsies per glomerular disease were compared using the four (seasons) × four (diseases) table chi-squared test. The differences in continuous and categorical variables were assessed using independent-samples t-test or Mann–Whitney U-test and chi-square test or Fisher’s exact test where appropriate, respectively. In addition, multiple comparisons were performed using analysis of variance and applying the Bonferroni correction.

A multivariable linear regression analysis that defined summer as the reference was constructed to identify the possible influence of seasonal variations on the severity of glomerular disease biopsy. In each analysis, the age, sex, and mean BP of the patients were treated as fixed covariates. Statistical significance was defined as a two-sided P-value of < 0.05. All statistical analyses were performed using SPSS v.25.0 (IBM Corp., Armonk, NY, USA).

### Ethical approval

All procedures performed in the studies involving human participants were in accordance with the ethical standards of the institutional and/or national research committee where the studies were conducted (IRB approval number: the Japanese Society of Nephrology, No. 79, September 2, 2019, and the Jikei University School of Medicine, 31–284(9783)) and per the principles of the 1964 Helsinki declaration and its later amendments or comparable ethical standards.

### Informed consent

Informed consent was obtained from all study participants or the parents if the participant is a child.

## Results

### Clinical characteristics

**Figure 2:**
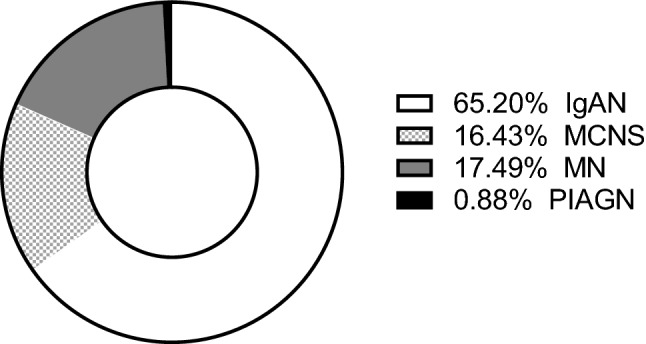
Frequency of the primary glomerular diseases. The number of renal biopsies was the highest in patients with IgAN (65.20%) and the lowest in patients with PIAGN (0.88%). IgA nephropathy (IgAN), minimal change nephrotic syndrome (MCNS), membranous nephropathy (MN), and postinfectious acute glomerulonephritis (PIAGN).

Table 1 summarizes the clinical characteristics of all the 13,989 patients at the time of their renal biopsy. Overall, the mean age of the patients was 43.8 ± 21.0 years (53.6% men). The mean eGFR was 74.6 ± 30.9 mL/min/1.73 m^2^. In total, 9,121 (65.2%) patients had IgAN, 2,298 (16.4%) had MCNS, 2,447 (17.5%) had MN, and 123 (0.88%) had PIAGN **(**Fig. 2**).**

**Table 1:** Background characteristics and laboratory parameters of the patients.

	Total
n	13,989
Age, mean (SD), years	43.8 ± 21.0
BMI, mean (SD), kg/m^2^	22.8 ± 4.5
Male (%)	53.6
Systolic blood pressure, mean (SD), mmHg	124.9 ± 18.7
Diastolic blood pressure, mean (SD), mmHg	74.5 ± 13.1
Mean blood pressure, mean (SD), mmHg	91.3 ± 13.8
TP, mean (SD), g/dL	6.3 ± 1.1
Alb, mean (SD), g/dL	3.5 ± 1.1
T-chol, mean (SD), mg/dL	241.5 ± 101.0
Creatinine, mean (SD), mg/dL	1.03 ± 1.02
eGFR, mean (SD), mL/min/1.73 m^2^	74.6 ± 30.9

BMI, body mass index; TP, total protein; Alb, albumin; T-cho, total cholesterol; eGFR, estimated glomerular filtration rate.

Values are expressed as mean ± standard deviation (SD).

### Seasonal variations in the number of renal biopsies

**Figure 3:**
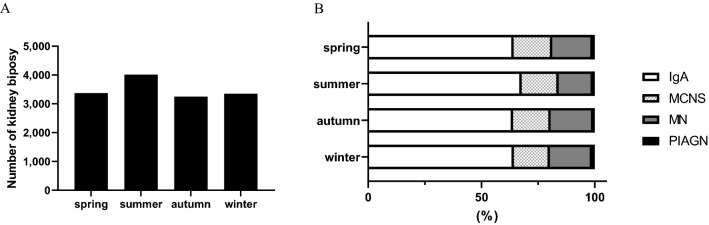
Seasonal variations in the number of renal biopsies. (**A**) Number of renal biopsies across the seasons. (**b**) Number of renal biopsies of the diseases across the seasons.

The number of kidney biopsies per season was as follows: 3,374 (24.1%) during spring, 4,015 (28.7%) during summer, 3,246 (23.2%) during autumn, and 3,354 (24.0%) during winter. The seasonal variation was significant (P < 0.001), and the number of renal biopsies was the highest during summer (Fig. 3a). Figure 3b displays the distribution of the number of renal biopsies across the seasons for the four glomerular diseases.

### Seasonal variations in IgAN

The seasonal variations in the biochemical and clinical parameters of the patients with IgAN are summarized in Table 2. The number of kidney biopsies was significantly (P < 0.001) higher during summer in patients with IgAN than those with other diseases. Significant seasonal differences were observed in age (P < 0.001), BMI (P < 0.001), systolic BP (P < 0.001), diastolic BP (P < 0.001), mean BP (P < 0.001), serum creatinine levels (P < 0.001), and UPE (P < 0.001), with the lowest peak values for these parameters occurring during summer. The distribution of age, UPE rates, and the CKD heat map categories significantly varied across the seasons. Cases involving younger patients were more common during summer compared with older patients, whereas adult cases were less common during summer and more common during autumn and winter. Regarding UPE, there were fewer cases involving severe proteinuria and more cases involving mild proteinuria during summer, contrary to the trend observed during winter. In the CKD heat map categories, the low-risk group was more common during summer and less common during autumn and winter. The high-risk and very high-risk groups were less common during summer.

**Table 2:** Seasonal changes in biochemical and clinical parameters in the patients with IgAN.

	Spring	Summer	Autumn	Winter	P value
N (%)	2,166 (23.7)	2,721 (29.8) ▲	2,076 (22.8)	2158 (23.7)	**-**
Age (year)	40.4 ± 17.9*	37.1 ± 18.2	41.4 ± 17.9*	41.3 ± 17.6*	** < 0.001**
< 18, n (%)	214 (20.9)	480 (47.0)▲	150 (14.7) ▽	178 (17.4) ▽	** < 0.001**
18–64, n (%)	1,697 (23.9)	2,000 (28.2)▽	1,672 (23.5)▲	1,731 (24.4)▲	
65 ≤ , n (%)	255 (25.5)	241 (24.1)	254 (25.4)	249 (24.9)	
Male, n (%)	1085 (50.1)	1364 (50.1)	1065 (51.3)	1123 (52.0)	0.484
BMI (kg/m^2^)	22.8 ± 4.1*	22.2** ± **4.1	22.9 ± 4.1*	22.8 ± 5.7*	** < 0.001**
Systolic BP (mmHg)	123.9 ± 17.8*	122.0 ± 17.7	125.3 ± 17.9*	125.2 ± 17.9*	** < 0.001**
Diastolic BP (mmHg)	74.3 ± 12.7*	72.9 ± 13.2	75.7 ± 13.1*	75.4 ± 13.1*	** < 0.001**
Mean BP (mmHg)	90.8 ± 13.2*	89.2 ± 13.7	92.3 ± 13.8*	91.0 ± 13.6*	** < 0.001**
Serum creatinine (mg/dL)	1.06 ± 1.03*	0.98 ± 0.76	1.08 ± 1.19*	1.09 ± 1.21*	** < 0.001**
eGFR (mL/min/1.73m^2^)	73.6 ± 30.3*	77.5 ± 30.6	71.8 ± 29.6*	71.9 ± 29.3*	** < 0.001**
UPE (g/day or g/gCr)	1.17 ± 1.69	1.11 ± 1.86	1.29 ± 1.81*	1.23 ± 1.59	** < 0.001**
< 0.15, n (%)	327 (24.1)	503 (37.1) ▲	273 (20.1)	252 (18.6) ▽	** < 0.001**
0.15–0.49, n (%)	541 (22.3)	752 (31.0)	550 (22.7)	580 (23.9)	
0.50 ≥ , n (%)	1,298 (24.3)	1,466 (27.4) ▽	1,253 (23.5)	1,326 (24.8) ▲	
Hematuria grade 2–3 n (%)	1,656 (23.8)	2,064 (29.6)	1,595 (22.9)	1,655 (23.7)	0.857
KDIGO prognosis risk of CKD, n (%)					
Very high risk	618 (23.7)	704 (27.0) ▽	624 (23.9)	665 (25.0)	** < 0.001**
High risk	819 (24.5)	922 (27.6) ▽	778 (23.3)	819 (24.5)	
Moderately increased risk	451 (22.3)	649 (32.1)	450 (22.3)	469 (23.2)	
Low risk	278 (24.1)	446 (38.7) ▲	224 (19.4) ▽	205 (17.8) ▽	

Data are presented as the mean ± SD or the frequency (percentage). Seasonal variations in the number of kidney biopsies per glomerular disease were examined using the four (seasons) × four (diseases) table chi-squared test. Chi-squared test with Bonferroni’s correction for multiple comparisons was also used for categorical variables. ▲ and ▽ show significantly higher and lower values than expected, respectively, in residual analyses using chi-squared tests. One-way analysis of variance with Tukey’s multiple comparison test was employed for continuous variables. P-values from chi-squared or Kruskal–Wallis test.

IgAN, immunoglobulin A nephropathy; BMI, body mass index; BP, blood pressure; eGFR, estimated glomerular filtration rate; UPE; urinary protein excretion.

*P < 0.05 versus summer.

### Seasonal variations in MCNS

Seasonal variations in the biochemical and clinical parameters of patients with MCNS are summarized in Table 3. Significant seasonal differences were detecetd in age (P < 0.001), BMI (P < 0.001), systolic BP (P < 0.001), diastolic BP (P < 0.001), mean BP (P < 0.001), serum creatinine levels (P < 0.01), and UPE (P < 0.001), with the lowest peak values for these parameters occurring during summer. The distribution of age, UPE rates, and CKD heat map categories significantly varied across the seasons. Cases involving younger patients were more common and during summer than those involving adult patients. Regarding UPE, there were fewer cases involving severe proteinuria and more cases involving mild proteinuria during summer, contrary to the trends observed during spring. In the CKD heat map categories, the low-risk group was more common during summer and less common during spring and winter, whereas the opposite trend was observed in the high-risk group.

**Table 3:** Seasonal differences in biochemical and clinical parameters in the patients with MCNS.

	Spring	Summer	Autumn	Winter	P value
N (%)	574 (25.0)	655 (28.5)	538 (23.4)	531 (23.1)	–
Age (year)	40.8 ± 24.2*	34.2 ± 24.5	38.9 ± 24.6*	38.8 ± 24.6*	** < 0.001**
< 18, n (%)	131 (20.4) ▽	241 (37.6) ▲	128 (20.0)	141 (22.0)	** < 0.001**
18–64, n (%)	299 (25.9)	296 (25.6) ▽	285 (24.7)	276 (23.8)	
65 ≤ , n (%)	144 (28.7)	118 (23.5) ▽	125 (24.9)	115 (22.9)	
Male, n (%)	338 (58.9)	378 (57.7)	298 (55.4)	314 (57.8)	0.583
BMI (kg/m^2^)	23.0 ± 4.5*	21.9 ± 4.7	22.9 ± 4.7*	22.8 ± 4.6*	** < 0.001**
Systolic BP (mmHg)	122.0 ± 18.9*	117.5 ± 17.6	121.6 ± 18.0*	122.2 ± 18.3*	** < 0.001**
Diastolic BP (mmHg)	73.0 ± 12.9*	70.4 ± 12.9	73.0 ± 12.5*	73.6 ± 12.6*	** < 0.001**
Mean BP (mmHg)	89.4 ± 13.8*	86.1 ± 13.4	89.2 ± 13.3*	89.8 ± 13.3*	** < 0.001**
Serum creatinine (mg/dL)	1.00 ± 1.01	0.87 ± 0.80	1.00 ± 0.97	0.92 ± 0.73	**0.002**
eGFR (mL/min/1.73m^2^)	81.5 ± 36.6*	88.8 ± 35.6	83.0 ± 37.0*	84.0 ± 36.9	** < 0.001**
UPE (g/day or g/gCr)	6.47 ± 6.60*	5.01 ± 5.81	5.90 ± 6.33	5.91 ± 6.20*	** < 0.001**
< 0.15, n (%)	94 (19.0) ▽	203 (41.1) ▲	104 (21.1)	93 (18.8)	** < 0.001**
0.15–0.49, n (%)	20 (23.8)	21 (25.0)	19 (22.6)	24 (28.6)	
0.50 ≥ , n (%)	460 (26.7) ▲	431 (25.1) ▽	415 (24.1)	414 (24.1)	
Hematuria grade 2–3 n (%)	199 (28.1)	180 (25.4)	167 (23.6)	163 (23.0)	0.06
KDIGO prognosis risk of CKD, n (%)					
Very high risk	159 (27.6)	147 (25.5)	136 (23.6)	135 (23.4)	** < 0.001**
High risk	302 (26.2)	288 (25.0) ▽	282 (24.4)	282 (24.4)	
Moderately increased risk	19 (23.8)	18 (22.5)	17 (21.3)	26 (32.5)	
Low risk	94 (19.3) ▽	202 (41.5) ▲	103 (21.1)	88 (18.1) ▽	

Data are presented as the mean ± SD or the frequency (percentage). Seasonal differences in the number of kidney biopsies per glomerular disease were assessed using the four (seasons) × four (diseases) table chi-squared test. For categorical variables, chi-squared test with Bonferroni's correction for multiple comparisons was also utilized. ▲ and ▽ show significantly higher and lower values than expected, respectively, in residual analyses using chi-squared tests. One-way analysis of variance with Tukey’s multiple comparison test was used for continuous variables. P-values from the chi-squared or Kruskal–Wallis test.

IgAN, immunoglobulin A nephropathy; BMI, body mass index; BP, blood pressure; eGFR, estimated glomerular filtration rate; UPE; urinary protein excretion.

*P < 0.05 versus summer.

### Seasonal variations in MN

Seasonal variations in the biochemical and clinical parameters of the patients with MN are summarized in Table 4. The number of kidney biopsies was significantly lower during summer in the patients with MN than those with other diseases. Significant seasonal differences were detected in systolic BP (P < 0.001), diastolic BP (P < 0.001), mean BP (P < 0.001), and UPE (P < 0.001), with the lowest peak values for these parameters occurring during summer. The distribution of age categories significantly varied across the seasons. Cases involving younger patients were more common during summer than during winter. In UPE and CKD heat map categories, no significant differences were found in the analyses of seasonal patterns.

**Table 4:** Seasonal variations in biochemical and clinical parameters in the patients with MN.

	Spring	Summer	Autumn	Winter	P value
N (%)	603 (24.6)	606 (24.8) ▽	611 (25.0)	627 (25.6)	–
Age (year)	64.2 ± 14.2	61.3 ± 17.8	64.3 ± 14.4	64.6 ± 13.6	0.079
<18, n (%)	11 (16.9)	38 (58.5) ▲	12 (18.5)	4 (6.2) ▽	** < 0.001**
18–64, n (%)	238 (24.6)	240 (24.8)	239 (24.7)	250 (25.9)	
65 ≤ n (%)	354 (25.0)	328 (23.2)	360 (25.4)	373 (26.4)	
Male, n (%)	349 (57.9)	376 (62.0)	359 (58.8)	376 (60.0)	0.482
BMI (kg/m^2^)	23.6 ± 3.7	23.5 ± 4.3	23.7 ± 3.9	23.5 ± 3.8	0.597
Systolic BP (mmHg)	132.6 ± 19.9*	128.7 ± 19.4	132.1 ± 19.6*	133.4 ± 20.6*	** < 0.001**
Diastolic BP (mmHg)	76.9 ± 13.4*	74.4 ± 12.6	77.0 ± 12.8*	77.9 ± 13.4*	** < 0.001**
Mean BP (mmHg)	95.4 ± 13.7*	92.5 ± 13.7	95.4 ± 13.3*	96.4 ± 14.3*	** < 0.001**
Serum creatinine (mg/dL)	1.02 ± 1.16	0.93 ± 1.10	0.94 ± 0.58	0.98 ± 0.62	0.305
eGFR (mL/min/1.73m^2^)	66.4 ± 24.3*	71.7 ± 26.9	68.4 ± 25.5	68.5 ± 25.3*	**0.041**
UPE (g/day or g/gCr)	5.02 ± 4.06	4.40 ± 3.82	5.17 ± 4.6*	5.08 ± 4.84*	**0.010**
< 0.15, n (%)	11 (26.8)	8 (19.5)	10 (24.4)	12 (29.3)	0.759
0.15–0.49, n (%)	29 (21.6)	41 (30.6)	32 (23.9)	32 (23.9)	
0.50 ≥ , n (%)	563 (24.8)	557 (24.5)	569 (25.0)	583 (25.7)	
Hematuria grade 2–3 n (%)	249 (24.5)	249 (24.5)	274 (26.9)	245 (24.1)	0.223
KDIGO prognosis risk of CKD, n (%)					0.765
Very high risk	234 (26.3)	207 (23.3)	218 (24.5)	230 (25.9)	
High risk	38 (23.9)	358 (25.3)	360 (25.4)	361 (25.5)	
Moderately increased risk	23 (21.3)	33 (30.6)	23 (21.3)	29 (26.9)	
Low risk	8 (24.2)	8 (24.2)	10 (30.3)	7 (21.2)	

Data are presented as the mean ± SD or the frequency (percentage). Seasonal variations in the number of kidney biopsies per glomerular disease were analyzed using the four (seasons) × four (diseases) table chi-squared test. Chi-squared test with Bonferroni’s correction for multiple comparisons was also employed for categorical variables. ▲ and ▽ indicate significantly higher and lower values than expected, respectively, in residual analyses utilizing chi-squared tests. One-way analysis of variance with Tukey’s multiple comparison test was used for continuous variables. P-values from the chi-squared or Kruskal–Wallis test.

IgAN, immunoglobulin A nephropathy; BMI, body mass index; BP, blood pressure; eGFR, estimated glomerular filtration rate; UPE; urinary protein excretion.

*P < 0.05 versus summer.

### Seasonal variations in PIAGN

Seasonal variations in the biochemical and clinical parameters of the patients with PIAGN are summarized in Table 5. Significant seasonal differences were detected in systolic BP (P < 0.028), diastolic BP (P < 0.036), mean BP (P < 0.02), serum creatinine levels (P < 0.03), and eGFR (P < 0.02). Serum creatinine levels during autumn were the highest, and the eGFR values during autumn were lower than those during spring. In the distributions of age, UPE rates, and CKD heat map categories, no significant differences were found during the analysis of seasonal patterns.

**Table 5:** Seasonal variations in biochemical and clinical parameters in the patients with PIAGN.

	Spring	Summer	Autumn	Winter	P value
N (%)	31 (25.2)	33 (26.8)	21 (17.1)	38 (30.9)	–
Age (year)	36.7 ± 23.4	46.5 ± 23.1	49.1 ± 25.2	49.3 ± 21.7	0.076
< 18, n (%)	8 (36.4)	4 (18.2)	4 (18.2)	6 (27.3)	0.747
18–64, n (%)	17 (25.4)	20 (29.9)	10 (14.9)	20 (29.9)	
65 ≤ , n (%)	6 (17.6)	9 (26.5)	7 (20.6)	12 (35.3)	
Male, n (%)	16 (51.6)	22 (66.7)	14 (66.7)	18 (47.4)	0.277
BMI (kg/m^2^)	22.7 ± 5.4	22.1 ± 4.2	25.4 ± 4.8	24.8 ± 6.5	0.078
Systolic BP (mmHg)	127.4 ± 28.2	137.9 ± 19.0	142.4 ± 13.6	135.0 ± 26.1	**0.028**
Diastolic BP (mmHg)	71.5 ± 15.6	80.5 ± 11.8	79.7 ± 13.6	75.8 ± 14.9	**0.036**
Mean BP (mmHg)	90.1 ± 18.2	99.6 ± 12.6	100.6 ± 11.7	95.5 ± 16.9	**0.02**
Serum creatinine (mg/dL)	1.47 ± 1.56**	1.50 ± 1.11**	3.54 ± 3.84	1.75 ± 1.59**	**0.03**
eGFR (mL/min/1.73m^2^)	66.0 ± 34.4	59.8 ± 33.8	39.1 ± 30.8***	49.0 ± 30.8	**0.02**
UPE (g/day or g/gCr)	1.97 ± 2.18	2.64 ± 2.87	3.67 ± 4.02	3.05 ± 3.59	0.38
< 0.15, n (%)	4 (30.8)	3 (23.1)	3 (23.1)	3 (23.1)	0.151
0.15–0.49, n (%)	7 (38.9)	8 (44.4)	1 (5.6)	2 (11.1)	
0.50 ≥ , n (%)	20 (21.7)	22 (23.9)	17 (18.5)	33 (35.9)	
Hematuria grade 2–3 n (%)	24 (23.1)	29 (27.9)	19 (18.3)	32 (30.8)	0.561
KDIGO prognosis risk of CKD, n (%)					
Very high risk	13 (18.3)	17 (23.9)	13 (18.3)	28 (39.4)	0.32
High risk	9 (33.3)	7 (25.9)	5 (18.5)	6 (22.2)	
Moderately increased risk	5 (31.3)	7 (43.8)	2 (12.5)	2 (12.5)	
Low risk	4 (44.4)	2 (22.2)	1 (11.1)	2 (22.2)	

Data are presented as the mean ± SD or frequency (percentage). Seasonal changes in the number of kidney biopsies per glomerular disease were compared using the four (seasons) × four (diseases) table chi-squared test. Chi-squared test with Bonferroni’s correction for multiple comparisons was also used for categorical variables. ▲ and ▽ revealed significantly higher and lower values than expected, respectively, in residual analyses using chi-squared tests. One-way analysis of variance with Tukey’s multiple comparison test was employed for continuous variables. P-values from the chi-squared or Kruskal–Wallis test.

IgAN, immunoglobulin A nephropathy; BMI, body mass index; BP, blood pressure; eGFR, estimated glomerular filtration rate; UPE; urinary protein excretion.

*P < 0.05 versus summer.

### Association between the seasons and the eGFR and UPE rates of the glomerular diseases

Table 6 shows the results of the multivariate linear regression analysis of eGFR and UPE rates without adjusting for the host factors. Both eGFR values and UPE rates were calculated with reference to summer as they tend to be lower during summer than during other seasons^27^. Multiple linear regression analyses revealed that summer was associated with higher eGFR in patients with IgAN, MCNS, and MN. Alternatively, proteinuria analyses indicated that summer was associated with low levels of proteinuria in patients with MCNS and MN. However, after adjusting for age, sex, BMI, and mean BP, multiple regression analyses revealed that spring (P = 0.038) and winter (P = 0.014) were associated with higher proteinuria in patients with MCNS and that spring (P = 0.036) was associated with lower eGFR compared with summer in patients with MN (Table7).

**Table 6:** Multivariable linear regression.

	IgAN				MCNS			
Dependent variables	eGFR		UPE		eGFR		UPE	
Seasons	Β (95% CI)	p	Β (95% CI)	p	Β (95% CI)	p	Β (95% CI)	p
Spring	− 0.055 (− 5.545 − − 2.161)	** < 0.001**	0.016 (− 0.035 − 0.163)	0.204	− 0.087 (− 11.414 − − 3.236)	** < 0.001**	0.078 (0.328 − 1.860)	**0.005**
Summer	Ref		Ref		Ref		Ref	
Autumn	− 0.080 (− 7.462 − − 4.038)	** < 0.001**	0.044 (0.082 − 0.281)	** < 0.001**	− 0.067 (− 9.919 − − 1.596)	**0.007**	0.052 (− 0.035 − 1.541)	0.061
Winter	− 0.079 (− 7.311 − − 3.924)	** < 0.001**	0.029 (0.021 − 0.219)	**0.017**	− 0.055 − 8.972 − − 0.620)	**0.024**	0.086 (0.461 − 2.033)	**0.002**
R^2^	0.006		0.001		0.005		0.005	

	MN				PIAGN			
Dependent variables	eGFR		UPE		eGFR		UPE	
Seasons	Β (95% CI)	p	Β (95% CI)	p	Β (95% CI)	p	Β (95% CI)	p
Spring	− 0.089 (− 8.088 − − 2.383)	** < 0.001**	0.060 (0.121 − 1.103)	**0.015**	0.080 (− 9.968 − 22.271)	0.451	− 0.092 (− 2.251 − 0.903)	0.399
Summer	Ref		Ref		Ref		Ref	
Autumn	− 0.056 (− 6.117 − − 0.431)	**0.024**	0.076 (0.278 − 1.257)	**0.002**	− 0.233 (− 38.668 − − 2.687)	**0.025**	0.121 (− 0.736 − 2.785)	0.251
Winter	− 0.074 (− 7.095 − − 1.446)	**0.003**	0.067 (0.188 − 1.161)	**0.007**	− 0.150 (− 26.185 – 4.486)	0.164	0.059 (− 1.094 − 1.906)	0.593
R^2^	0.005		0.004		0.058		0.008	

IgAN, immunoglobulin A nephropathy; MCNS, minimal change nephrotic syndrome; MN, membranous nephropathy; PIAGN, post-infectious acute glomerulonephritis; eGFR, estimated glomerular filtration rate; UPE; urinary protein excretion.

**Table 7:** Multivariable linear regression (all analyses were adjusted for age, sex, BMI and MBP).

	IgAN				MCNS			
Dependent variables	eGFR		UPE		eGFR		UPE	
Seasons	Β (95% CI)	p	Β (95% CI)	p	Β (95% CI)	p	Β (95% CI)	p
Spring	0.002 (− 1.083 − 1.366)	0.821	− 0.006 (− 0.118 − 0.072)	0.636	0.009 (− 2.102 − 3.559)	0.614	0.057 (0.046 − 1.562)	**0.038**
Summer	Ref		Ref		Ref		Ref	
Autumn	− 0.004 (− 1.5083 − 0.979)	0.676	0.013 (− 0.044 − 0.149)	0.282	0.005 (− 2.447 − 3.298)	0.771	0.030 (− 0.338 − 1.219)	0.267
Winter	− 0.006 (− 1.644 − 0.813)	0.507	0.002 (− 0.087 − 0.103)	0.867	0.014 (− 1.664 − 4.109)	0.406	0.067 (0.193—1.747)	**0.014**
R^2^	0.486		0.070		0.533		0.043	

	MN				PIAGN			
Dependent variables	eGFR		UPE		eGFR		UPE	
Seasons	Β (95% CI)	p	Β (95% CI)	p	Β (95% CI)	p	Β (95% CI)	p
Spring	− 0.044 (− 4.972 − − 0.168)	**0.036**	0.034 (− 0.136 − 0.819)	0.161	− 0.006 (− 13.990 − 13.126)	0.950	0.051 (− 1.210 − 1.962)	0.640
Summer	Ref		Ref		Ref		Ref	
Autumn	− 0.007 (− 2.809 − 1.982)	0.735	0.046 (− 0.016 − 0.936)	0.058	− 1.881 (− 28.492 − 0.736)	0.062	0.144 (− 0.491 − 2.928)	0.161
Winter	− 0.019 (− 3.485 − 1.282)	0.365	0.036 (− 0.112 − 0.835)	0.134	0.876 (− 18.500 − 7.151)	0.383	0.126 (− 0.630 − 2.370)	0.253
R^2^	0.308		0.076		0.405		0.122	

BMI, body mass index; MBP, mean blood pressure IgAN, immunoglobulin A nephropathy; MCNS, minimal change nephrotic syndrome; MN, membranous nephropathy; PIAGN, post-infectious acute glomerulonephritis; eGFR, estimated glomerular filtration rate; UPE; urinary protein excretion.

## Discussion

Furthermore, we examined whether seasonal variatoins affected the clinical characteristics of the patients at the time of biopsy.

The overall analysis revealed that the number of renal biopsies was significantly higher during summer, particularly for patients with IgAN. This may be because school urinalysis and physical examinations are often conducted during spring in Japan^28^, and admissions for kidney biopsies are more common during the summer holidays than during the other periods of the year. Thus, it is reasonable to assume that IgAN and MCNS are more common in younger individuals presenting with mild cases during summer, similar to the results of the present study.

The analyses of seasonal variations in patients with IgAN in this study revealed a significant increase in the number of renal biopsies in adult patients during autumn and winter. The number of patients with severe proteinuria was particularly high during winter. Previous retrospective studies involving patients with IgAN have reported high proteinuria exacerbation during autumn and winter^29^. Similarly, patients with diabetic nephropathy and pediatric MCNS are reportedly more prone to proteinuria and albuminuria exacerbations during autumn and winter compared with summer. The mechanism underlying this worsening of proteinuria during autumn and winter is not well understood; however, the lack of seasonal variations in the degree of occult urine suggests that age, BP, and renal function may be responsible for the worsening of proteinuria^30^. BP rises during winter owing to vasoconstriction and falls during summer because of vasodilation due to temperature changes^31^. Our results also indicate that BP increases in autumun and winter, which supports the results reported in previous studies^3^. However, differences in BP values are trivial and may not be clinically important. Furthermore, other unknown factors may be involved. In addition, IgAN may be associated with the exacerbation of proteinuria due to preceding upper respiratory tract infections, which tends to occur more frequently during winter^18^.

The seasonal variation in the patients with MCNS in this study was characterized by a higher number of renal biopsies during summer in young people than in adults. Regarding proteinuria, there were more cases of mild proteinuria during summer and more cases of severe proteinuria during spring than during the other seasons. Several previous studies have reported seasonal variations in patients with pediatric MCNS characterized by a peak during autumn for primary MCNS cases and a peak during spring for recurrent MCNS cases, suggesting an association with the amount of mite allergen in hay fever and house dust^17,18^. A previous study reported that prior respiratory viral infections are associated with recurrent nephrotic syndrome, irrespective of the type of virus^32^. Herein, no exacerbation of proteinuria was observed during autumn; however, proteinuria exacerbated during spring, as reported previously, suggesting some type of spring allergic factor.

Our analyses did not detect any significant seasonal variations in the patients with MN and PIAGN. To the best of our knowledge, there have been no reports regarding seasonal variations in patients with MN, and although our study suggests that renal dysfunction is more common during spring than during the other seasons, it was difficult to identify a mechanism or hypothesis to support this finding. For PIAGN, a causal relationship with streptococcal infections has been suggested, which increases the number of cases and exacerbates the disease during winter^32,33^. However, the results of the present study did not appear to be statistically significant owing to the small number of study participants^3^. This may be due to the low absolute number of PIAGN cases in our study owing to the widespread use of antibiotics in recent years.

Notably, the clinical features of the disease are influenced by various factors and not only seasonal variations. Therefore, we assessed seasonal variations in clinical features adjusted for age, sex, body size, and BP. Notably, when adjusted for the abovementioned factors, the seasonal factor was weaker in all the glomerular diseases, and only MCNS exhibited proteinuria exacerbation during spring and winter. These results support previous evidence stating that certain infectious or allergic factors contribute to MCNS exacerbation during spring and winter^16,17^.

Previous studies have reported that ambient temperature, humidity, and air pollution (NO_2_, SO_2_, and PMs) with seasonal variations can cause dehydration and abnormal immune responses that have been associated with the risk of developing urolithiasis, acute kidney injury, CKD, and urinary tract infections^34,35^. Although, to the best of our knowledge, no studies have yet demonstrated an association between primary glomerular diseases and these external factors, it is possible that these factors have influenced the seasonal variation observed in this study.

This study has several limitations. First, it was a national study, and thus, its findings cannot be internationally generalized. Second, the institutions providing data to the J-RBR are not evenly distributed throughout Japan. Third, as the analysis was based on renal biopsy data, there are no uniform indications for renal biopsies, which may have caused sampling bias and data collection not reflecting disease incidence and severity. Moreover, location-specific data, such as temperature and regional differences, were not assessed. Further studies are warranted regarding the time of disease onset and treatment initiation to clarify the strong causal role of seasonal factors in the development of glomerular diseases.

Although there are various limitations in exploring the mechanisms and causes of seasonal variations in glomerular diseases, the greatest strength of this study is the large sample of patients collected from multiple facilities across Japan.

In conclusion, the results of this study demonstrate seasonal variations in the number of renal biopsies in Japan and indicate that seasonal variations in disease severity exist, particularly in patients with IgAN and MCNS. In both cases, the number of kidney biopsies increases during summer. In patients with IgAN, increase in the number of severe cases during winter may be largely owing to age and BP. Conversely, increase in the number of severe cases of patients with MCNS during winter may be due to seasonal variations. Understanding the seasonal variations in the number of renal biopsies will help establish a better presystem for renal biopsy decision and help identify triggers in the patient background, such as BP, lifestyle, infections, and allergies. Furthermore, our findings may provide important insights for the future understanding of the pathophysiology of primary glomerular diseases.

## Supplementary Information

Supplementary Information.

## Data Availability

These data in the present study are available from the corresponding author upon reasonable request.
