# Supplementary material for: Seasonal variations in renal biopsy numbers and primary glomerular disease features based on the Japan renal biopsy registry
Source: Sci Rep. 2023 Mar 29;13:5123. doi: 10.1038/s41598-023-32182-7 (PMC10060207; doi:10.1038/s41598-023-32182-7)
Supplement: Supplementary file 1 — Supplementary Information. [file 41598_2023_32182_MOESM1_ESM.docx]

**Supplementary Information**

The following investigators and initial institutions have participated in the development of the J-RBR since 2007: Hirofumi Makino and Hitoshi Sugiyama (Okayama University), *late* Takashi Taguchi (Nagasaki University), Hitoshi Yokoyama (Kanazawa Medical University), Hiroshi Sato (Tohoku University; present institution: JR Sendai Hospital), Takao Saito (Fukuoka University; present institution: Sanko Clinic), Yoshie Sasatomi (Fukuoka University; present institution: Kanenokuma Hospital), Yukimasa Kohda (Kumamoto University; present institution: Hikarinomori Clinic), Shinichi Nishi (Niigata University; present institution: Kobe University), Kazuhiko Tsuruya (Kyushu University; present institution: Nara Medical University), Yutaka Kiyohara (Kyushu University; present institution: Hisayama Research Institute for Lifestyle Diseases), Hideyasu Kiyomoto (Kagawa University; present institution: Tohoku Medical Megabank Organization, Tohoku University), Hiroyuki Iida (Toyama Prefectural Central Hospital; present institution: Toyama Prefectural Rehabilitation Hospital), Tamaki Sasaki (Kawasaki Medical School), *late* Makoto Higuchi (Shinshu University), Motoshi Hattori (Tokyo Women’s Medical University), Kazumasa Oka (Osaka Kaisei Hospital; present institution: Hyogo Prefectural Nishinomiya Hospital), Shoji Kagami (The University of Tokushima Graduate School), Michio Nagata (University of Tsukuba), Tetsuya Kawamura (The Jikei University School of Medicine), Masataka Honda (Tokyo Metropolitan Children’s Medical Center), Yuichiro Fukasawa (KKR Sapporo Medical Center; present institution: Sapporo City General Hospital), Atsushi Fukatsu (Kyoto University Graduate School of Medicine; present institution: Fukatsu Medical Clinic), Kunio Morozumi (Japanese Red Cross Nagoya Daini Hospital; present institution: Masuko Memorial Hospital), Norishige Yoshikawa (Wakayama Medical University; present institution: Takatsuki General Hospital), Yukio Yuzawa (Fujita Health University), Seiichi Matsuo (Nagoya University) and Kensuke Joh (Chiba-East National Hospital; present institution: The Jikei University School of Medicine).

**Hokkaido District**

・Asahikawa Medical University Hospital (Division of Cardiology, Nephrology, Pulmonology and Neurology, Department of Internal Medicine), Naoyuki Hasebe, Naoki Nakagawa

・National Hospital Organization Hokkaido Medical Center (Department of Nephrology), Sekiya Shibazaki, Tomotsune Miyamoto, Masanori Ito

・Hokkaido University Graduate School of Medicine (Department of Rheumatology, Endocrinology and Nephrology, Faculty of Medicine and Graduate School of Medicine, Hokkaido University), Saori Nishio, Daigo Nakazawa

・Hokkaido University Graduate School of Medicine (Department of Pediatrics), Takayuki Okamoto, Yasuyuki Sato

・KKR Sapporo Medical Center (Department of Pathology), Akira Suzuki

・Sapporo Medical University (Department of Cardiovascular, Renal and Metabolic Medicine), Tomohisa Yamashita, Marenao Tanaka

・Sapporo City General Hospital, Yuichiro Fukasawa

・Teine Keijinkai Hospital (Department of Nephrology), Hideki Takizawa, Norihito Moniwa

**Tohoku District**

・Iwate Prefectural Central Hospital (Department of Nephrology and Rheumatology), Jun Soma, Izaya Nakaya

・Fukushima Medical University (Department of Nephrology and Hypertension), Junichiro James Kazama, Kenichi Tanaka, Mizuko Tanaka

・Japan Community Health care Organization Sendai Hospital (Department of Nephrology), Mitsuhiro Sato, Satoru Sanada

・Tohoku University Hospital and affiliated hospitals (Internal Medicine), Mariko Miyazaki, Tasuku Nagasawa, Koji Okamoto

・Yamagata University School of Medicine (Department of Cardiology, Pulmonology, and Nephrology), Tsuneo Konta, Kazunobu Ichikawa

・Yamagata University School of Medicine (Department of Pediatrics), Daisuke Ogino

**Kanto District**

・National Hospital Organization Chibahigashi National Hospital (Department of Pathology), Hiroshi Kitamura, (Department of Nephrology), Toshiyuki Imasawa, (Department of Pediatrics), Chieko Matsumura, (Department of Surgery), Naotake Akutsu

・National Hospital Organization Chiba-East Hospital (Department of Urology), Koichi Kamura (*) *present address, Harunclinic Sakura

・Dokkyo Medical University Saitama Medical Center (Department of Nephrology), Tetsuro Takeda

・Dokkyo Medical University (Department of Nephrology and Hypertension ), Toshihiko Ishimitsu

・Gunma University Graduate School of Medicine (Department of Nephrology and Rheumatology), Keiju Hiromura, Yoriaki Kaneko, Hidekazu Ikeuchi, Toru Sakairi

・Jichi Medical University (Division of Nephrology), Daisuke Nagata, Osamu Saito, Tetsu Akimoto

・Jichi Medical University, Saitama Medical Center (Division of Nephrology, Department of Integrated Medicine), Yoshiyuki Morishita

・The Jikei University School of Medicine (Division of Nephrology and Hypertension), Takashi Yokoo, Nobuo Tsuboi, Hiroyuki Ueda, Kentaro Koike, Go Kanzaki

・The Jikei University School of Medicine, Katsushika Medical Center (Division of Nephrology and Hypertension), Yudo Tanno, Shinya Yokote

・The Jikei University School of Medicine, Daisan Hospital (Division of Nephrology and Hypertension), Yoichi Miyazaki, Masahiro Okabe

・The Jikei University Kashiwa Hospital (Division of Nephrology and Hypertension), Masato Ikeda, Akihiro Shimizu

・Juntendo University Faculty of Medicine (Department of Nephrology), Yusuke Suzuki, Tomohito Goda, Masao Kihara, Miyuki Takagi

・Japanese Red Cross Ashikaga Hospital (Department of Nephrology), Keita Hirano

・Kawaguchi Municipal Medical Center (Division of Nephrology), Masahiro Ishikawa

・Kyorin University School of Medicine (Department of Nephrology and Rheumatology), Shinya Kaname, Kazuhito Fukuoka, Takahisa Kawakami

・Mito Saiseikai General Hospital (Division of Nephrology), Itaru Ebihara, Chihiro Sato

・Nippon Medical School (Division of Nephrology, Department of Internal Medicine), Yukinao Sakai, Akio Hirama, Akiko Mii

・Nihon University School of Medicine (Division of Nephrology, Hypertension and Endocrinology), Yoshinobu Fuke, Masanori Abe

・Saitama Medical University, Faculty of Medicine (Department of Nephrology), Hirokazu Okada, Tsutomu Inoue

・Saitama Medical University, Saitama Medical Center (Department of Nephrology and Hypertension), Takatsugu Iwashita, Akito Maeshima, Hajime Hasegawa

・Saiyu Soka Hospital（Department of Internal Medicine), Masamitsu Ubukata

・Showa University School of Medicine (Division of Nephrology, Department of Medicine), Masayuki Iyoda, Takanori Shibata

・Showa University Fujigaoka Hospital (Division of Nephrology, Department of Medicine), Yoshihiko Inoue

・St. Marianna University School of Medicine (Division of Nephrology and Hypertension, Department of Internal Medicine), Tomo Suzuki, Daisuke Ichikawa, Sayuri Shirai, Yugo Shibagaki

・Tokai University School of Medicine (Division of Nephrology, Endocrinology and Metabolism), Takehiko Wada, Masafumi Fukagawa

・Teikyo University School of Medicine (Department of Internal Medicine), Yoshihide Fujigaki

・Teikyo University School of Medicine (Department of Urology), Shigeo Horie(*), Satoru Muto(*) *present address, Juntendo University School of Medicine (Department of Urology)

・Tokyo Medical University Ibaraki Medical Center (Department of Nephrology), Masaki Kobayashi, Kouichi Hirayama, Homare Shimohata

・Tokyo Metropolitan Children's Medical Center (Department of Nephrology), Riku Hamada (Department of General Pediatrics), Hiroshi Hataya

・Tokyo Women's Medical University (Department of Pediatric Nephrology), Motoshi Hattori, Kenichiro Miura, Kiyonobu Ishizuka, Naoto Kaneko

・Tokyo Women's Medical University (Department of Nephrology), Kosaku Nitta, Keiko Uchida, Takahito Moriyama

・Toranomon Hospital, Nephrology Center, Yoshifumi Ubara, Tatsuya Suwabe, Junichi Hoshino, Noriko Hayami

・The University of Tokyo (Department of Nephrology and Endocrinology), Masaomi Nangaku, Tetsuhiro Tanaka, Yoshifumi Hamasaki, Kenjiro Honda

・The University of Tokyo(Department of Pediatrics), Yutaka Harita, Shoichiro Kanda, Yuko Kajiho

・University of Tsukuba (Department of Nephrology), Kunihiro Yamagata, Joichi Usui, Tetsuya Kawamura

・Yokohama City University Graduate School of Medicine (Department of Medical Science and Cardiorenal Medicine), Kouichi Tamura, Hiromichi Wakui, Tomohiko Kanaoka, Ryu Kobayashi

・Yokohama City University Medical Center, Nobuhito Hirawa, Sanae Saka, Akira Fujiwara

**Koushinetsu District**

・Niigata University Graduate School of Medical and Dental Sciences (Division of Clinical Nephrology and Rheumatology), Ichiei Narita, Shin Goto, Yumi Itoh, Naofumi Imai

・Shinshu University School of Medicine (Department of Nephrology), Yuji Kamijo, Koji Hashimoto, Akinori Yamaguchi, Harada Makoto

・University of Yamanashi Hospital (Third Department of Internal Medicine), Kazuya Takahashi, Fumihiko Furuya

**Hokuriku District**

・National Hospital Organization Kanazawa Medical Center (Department of Nephrology and Rheumatology), Kiyoki Kitagawa

・Kanazawa Medical University School of Medicine (Department of Nephrology), Hitoshi Yokoyama, Kengo Furuichi, Keiji Fujimoto, Norifumi Hayashi

・Kanazawa Medical University (Department of Diabetology & Endocrinology), Daisuke Koya, Munehiro Kitada, Yuka Kuroshima

・Kanazawa University Hospital (Division of Nephrology), Takashi Wada, Miho Shimizu, Norihiko Sakai, Yasunori Iwata

・Komatsu Sophia Hospital, Yasuhiro Katou, Yuta Yamamura

・Koshino Internal Medicine Clinic, Yoshitaka Koshino

・Public Central Hospital of Matto-Ishikawa, Chikako Takaeda

・Sugita Genpaku Memorial Obama Municipal Hospital, Haruyoshi Yoshida, Takayasu Horiguchi

・Toyama Prefectural Central Hospital (Department of Internal Medicine), Yasuyuki Shinozaki, Masahiko Kawabata

・Toyama City Hospital (Department of Internal Medicine), Satoshi Ota, Yoh-ichi Ishida

・University of Fukui, Faculty of Medical Sciences (Department of Nephrology), Masayuki Iwano, Naoki Takahashi, Kenji Kasuno, Daisuke Mikami

・University of Toyama (Second Department of Internal Medicine), Hidenori Yamazaki

**Tokai District**

・Aichi Children's Health and Medical Center (Department of Pediatric Nephrology), Naoya Fujita, Satoshi Hibino, Kazuki Tanaka

・Aichi Medical University School of Medicine (Division of Nephrology and Rheumatology), Yasuhiko Ito, Takuhito Nagai, Takayuki Katsuno, Hironobu Nobata

・Chuno Kosei Hospital, Shogo Kimura, Yuka Soga

・Fujinomiya City General Hospital, Masanori Sakakima

・Fujita Health University School of Medicine (Department of Nephrology), Yukio Yuzawa, Naotake Tsuboi, Hiroki Hayashi, Kazuo Takahashi

・Hamamatsu University School of Medicine, University Hospital (Internal Medicine1, Division of Nephrology), Hideo Yasuda, Naro Ohashi, Taichi Sato

・Japanese Red Cross Nagoya Daini Hospital (Kidney Center), Asami Takeda, Yasuhiro Otsuka

・Nagoya City University East Medical Center, Taisei Suzuki, Minami Mizutani

・Nagoya City University Graduate School of Medical Sciences (Department of Cardio-Renal Medicine and Hypertension), Michio Fukuda, Masashi Mizuno, Satoru Kominato

・Nagoya Kyoritsu Hospital (Department of Internal Medicine), Hirotake Kasuga

・Nagoya University Graduate School of Medicine (Department of Nephrology), Shoichi Maruyama, Yoshinari Yasuda, Tomoki Kosugi, Takuji Ishimoto

・Shizuoka General Hospital (Department of Nephrology), Noriko Mori, Satoshi Tanaka

・Mie University Graduate School of Medicine (Department of Cardiology and Nephrology), Tomohiro Murata, Mika Fujimoto, Kan Katayama

・Japan Community Health care Organization Yokkaichi Hazu Medical Center (Division of Nephrology and Blood Purification), Yasuhide Mizutani, Masato Miyake, Shunpei Nawa

**Kinki District**

・Hyogo Prefectural Nisihinomiya Hospital (Department of Pathology), Kazumasa Oka

・Hyogo Prefectural Kobe Children's Hospital (Department of Nephrology), Hiroshi Kaito

・Ikeda City Hospital (Department of Nephrology), Nobuyuki Kajiwara

・Kitano Hospital, Tazuke Kofukai Medical Research Institute (Department of Nephrology and Dialysis), Tatsuo Tsukamoto, Tomomi Endo, Eri Muso

・Kobe University Graduate School of Medicine (Division of Nephrology and Kidney Center), Shinichi Nishi, Shunsuke Goto

・Kobe University Graduate School of Medicine (Department of Pediatrics), Kazumoto Iijima, Kandai Nozu, Tomoko Horinouchi

・Japan Community Health care Organization Kobe Central Hospital, Yoko Adachi, Takaaki Nishihara, Michitsugu Kamezaki

・National Hospital Organization Kyoto Medical Center (Division of Nephrology), Koichi Seta

・Kyoto Prefectural University of Medicine Graduate School of Medical Science (Department of Nephrology), Keiichi Tamagaki, Tetsuro Kusaba, Yayoi Shiotsu

・Kyoto University Graduate School of Medicine (Department of Nephrology), Motoko Yanagita, Hideki Yokoi, Kaoru Sakai, Akira Ishii

・Nara Medical University (Department of Nephrology), Kazuhiko Tsuruya, Kenichi Samejima

・National Cerebral and Cardiovascular Center (Division of Hypertension and Nephrology), Fumiki Yoshihara

・National Hospital Organization Osaka National Hospital (Department of Nephrology), Hirotsugu Iwatani

・Osaka City University Graduate School of Medicine (Department of Nephrology), Katsuhito Mori, Akihiro Tsuda, Shinya Nakatani

・Osaka City General Hospital (Division of Nephrology and Hypertension), Yoshio Konishi, Takashi Morikawa, Chizuko Kitabayashi

・Osaka City General Hospital (Division of Pediatrics), Rika Fujimaru

・Osaka General Medical Center (Department of Kidney Disease and Hypertension), Terumasa Hayashi, Tatsuya Shoji

・Osaka Women's and Children's Hospital (Department of Pediatric Nephrology and Metabolism), Katsusuke Yamamoto

・Osaka Medical College (Department of Pediatrics), Akira Ashida

・Osaka Red Cross Hospital (Department of Nephrology), Akira Sugawara, Masao Koshikawa, Yoshihisa Ogawa, Tomoko Kawanishi

・Osaka Rosai Hospital (Department of Nephrology), Atsushi Yamauchi, Katsuyuki Nagatoya, Daisuke Mori, Ryota Haga

・Osaka University Graduate School of Medicine (Department of Nephrology), Yoshitaka Isaka, Ryohei Yamamoto, Tomoko Namba-Hamano

・Saiseikai Shiga Hospital (Division of Nephrology), Toshiki Nishio

・Shiga University of Medical Science (Department of Medicine), Shinichi Araki

・Shirasagi Hospital (Kidney Center), Shigeichi Shoji, Kenjiro Yamakawa, Senji Okuno

・Toyonaka Municipal Hospital (Division of Nephrology), Megumu Fukunaga

・Wakayama Medical University (Department of Pediatrics), Yuko Shima,

・Wakayama Medical University (Department of Nephrology), Takashi Shigematsu, Masaki Ohya

**Chugoku District**

・Kawasaki Medical School (Department of Nephrology and Hypertension), Naoki Kashihara, Tamaki Sasaki, Hajime Nagasu

・Kurashiki Central Hospital (Division of Nephrology), Kenichiro Asano, Motoko Kanzaki, Kosuke Fukuoka

・Hiroshima University Hospital (Department of Nephrology), Takao Masaki, Shigehiro Doi, Ayumu Nakashima, Toshiki Doi

・Mizushima Kyodo Hospital (Department of Nephrology), Kan Yamazaki, Nobuyoshi Sugiyama, Yuichiro Inaba, Kouji Ozeki

・Okayama Saiseikai General Hospital (Department of Nephrology), Makoto Hiramatsu, Keisuke Maruyama, Noriya Momoki

・Okayama University Graduate School of Medicine, Dentistry and Pharmaceutical Sciences (Department of Nephrology, Rheumatology, Endocrinology and Metabolism), Jun Wada, Hiroshi Morinaga, Ayu Akiyama, Yasuhiro Onishi

・Okayama University Graduate School of Medicine, Dentistry and Pharmaceutical Sciences (Department of Pediatrics), Hiroyuki Miyahara

・Saiseikai Yamaguchi General Hospital (Department of Internal Medicine), Tsuyoshi Imai

・Shimane University Faculty of Medicine (Division of Nephrology), Takafumi Ito, Masahiro Egawa, Shohei Fukunaga

・Tottori University, Faculty of Medicine (Division of Pediatrics and Perinatology), Shinichi Okada, Koichi Kitamoto, Hiroki Yokoyama, Yuko Yamada

**Shikoku District**

・Kagawa University, Faculty of Medicine (Department of Cardiorenal and Cerebrovascular Medicine & Department of Clinical Pathology), Tadashi Sofue, Tetsuo Minamino, Emi Ibuki

・Kochi University, Kochi Medical School (Department of Endocrinology, Metabolism and Nephrology), Yoshio Terada, Taro Horino, Yoshiko Shimamura,Tatsuki Matsumoto

・Kochi University, Kochi Medical School (Department of Pediatrics), Mikiya Fujieda, Masayuki Ishihara, Yoshiki Nagao

・Tokushima University Graduate School (Department of Pediatrics, Institute of Biomedical Sciences), Shoji Kagami, Maki Urushihara, Yukiko Kinoshita

・Tokushima University Graduate School (Department of Nephrology, Institute of Biomedical Sciences), Hideharu Abe, Kojiro Nagai

**Kyushu District**

・Fukuoka University (Division of Nephrology and Rheumatology, Department of Internal Medicine, Faculty of Medicine), Kosuke Masutani, Tetsuhiko Yasuno, Kenji Ito

・Japanese Red Cross Fukuoka Hospital (Department of Pediatrics), Ken Hatae, Manao Nishimura, Hiroyo Maruyama, Miyu Aoyama

・Japanese Red Cross Fukuoka Hospital (Nephrology and Dialysis Center), Koji Mitsuiki

・Kumamoto University Graduate School of Medical Sciences (Department of Nephrology), Masashi Mukoyama, Masataka Adachi

・Kurume University School of Medicine (Division of Nephrology, Department of Medicine), Kei Fukami, Junko Yano

・Kyushu University Graduate School of Medical Sciences (Department of Medicine and Clinical Science), Toshiaki Nakano, Akihiro Tsuchimoto, Yuta Matsukuma, Kenji Ueki

・Kyushu University Graduate School of Medical Sciences (Department of Environmental Medicine), Yutaka Kiyohara, Toshiharu Ninomiya, Masaharu Nagata

・Miyazaki Prefectural Miyazaki Hospital (Division of Nephrology), Naoko Yokota-Ikeda, Keiko Kodama

・Nagasaki University Hospital (Department of Pathology), *late* Takashi Taguchi

・Nagasaki University Hospital (Department of Nephrology), Tomoya Nishino, Kenta Torigoe, Kiyokazu Tsuji

・National Hospital Organization Fukuokahigashi Medical Center (Division of Nephrology), Yusuke Kuroki

・National Hospital Organization Kyushu Medical Center, Masaru Nakayama

・Oitaken Kouseiren Tsurumi Hospital (Division of Nephrology), Ryokichi Yasumori

・Saga University, Faculty of Medicine (Department of Internal Medicine), Motoaki Miyazono, Tsuyoshi Takashima, Shuichi Rikitake, Makoto Fukuda

・St. Mary's Hospital, Harumichi Higashi

・University of Miyazaki Hospital (Division of Nephrology), Shouichi Fujimoto, Masao Kikuchi, Shoko Ochiai

・University of Occupational and Environmental Health (Second Department of Internal Medicine), Masahito Tamura, Tetsu Miyamoto

・University of the Ryukyus Graduate School of Medicine (Department of Cardiology, Nephrology and Neurology), Yusuke Ohya, Kentaro Kohagura
